# Alexithymia in Young Adults With Substance Use Disorders: Critical Issues About Specificity and Treatment Predictivity

**DOI:** 10.3389/fpsyg.2018.00645

**Published:** 2018-05-22

**Authors:** Micol Parolin, Marina Miscioscia, Pietro De Carli, Patrizia Cristofalo, Michela Gatta, Alessandra Simonelli

**Affiliations:** ^1^Department of Developmental Psychology and Socialization, University of Padua, Padua, Italy; ^2^Department of Women's and Children's Health, University of Padua, Padua, Italy; ^3^Therapeutic Community “Villa Renata”, Venice, Italy; ^4^Childhood Adolescence Family Unit, Ulss6 Veneto, Padua, Italy

**Keywords:** alexithymia, substance use disorder, young adulthood, treatment outcome, 20 Item -Toronto Alexithymia Scale

## Abstract

Several studies have reported high rates of alexithymia in drug-dependent individuals, but supporting evidence attests association between alexithymia and a variety of psychiatric disorders, raising doubts about its specificity. Moreover, controversies are emerging about alexithymia assessment: self-report measures present shortcomings with respect to discriminant validity and reliability. As regards treatment for substance use disorders (SUDs), alexithymia has been linked to poorer outcomes, but the results are inconsistent. The aim of the present study is to investigate alexithymia in substance-dependent young adults by examining: (a) the specificity of alexithymia in drug-dependent inpatients, compared to healthy individuals and patients with psychiatric disorders (behavioral and emotional disorders) and (b) the predictivity of alexithymia in determining treatment outcomes in terms of relapses, drop-outs from treatment and the rate of relapse per month of treatment. Two studies were conducted to fulfill these aims: Study 1 and Study 2. Study 1 involved 90 late adolescents, aged 17–21. To fulfill the first aim, 30 inpatients diagnosed with SUD were compared with 30 healthy controls and 30 individuals referred to an outpatient neuropsychiatric unit (a). The participants completed the Toronto Alexithymia Scale−20 (TAS-20) and the Symptom Checklist-90-Revised (SCL-90-R). The results indicated that both clinical groups reported higher TAS-20 scores than the non-clinical subjects, but they did not differ from each other (a); moreover, a large correlation was detected between alexithymia and depressive symptoms, as assessed by the SCL-90-R. Study 2 involved 55 inpatients with SUD recruited in a therapeutic community. The participants completed the TAS-20, and clinicians filled out the Observer Alexithymia Scale (OAS). No association was found between self-report and observational measures. Neither self-reported nor observed alexithymia predicted the number of relapses, drop-out from treatment, or the rate of relapses per month of treatment (b). When the interaction with gender was explored, the global score of alexithymia and the “Distant” OAS subscale predicted the number rate relapses only in males. The TAS-20 did not discriminate between the clinical groups. The limited ability of both observed and self-reported measures in predicting treatment outcome raises questions on the specificity of alexithymia among the substance-dependent inpatient population.

## Theoretical background

Alexithymia refers to the psychological dysfunctional trait of having no words to express emotions or feelings (Sifneos, 1973). It is a multidimensional construct comprising emotional and cognitive components: difficulties in identifying and describing feelings as well as in differentiating somatic sensations and feelings, lack of fantasy, and imagination and an externally oriented cognitive style (Nemiah and Sifneos, 1970; Nemiah et al., 1976; Taylor et al., 1997). Interestingly, the relevance of alexithymia has grown exponentially in the last decades; it is currently considered a relevant concept for a range of psychological and physical disorders (Taylor et al., 1997; Taylor and Bagby, 2004), and attention is paid to its relationships with other constructs, such as emotional intelligence, negative affect, and its role in predicting treatment outcomes (Morie et al., 2016).

### Alexithymic traits in drug-dependent individuals

Vast research has suggested that alexithymia is quite common in patients with substance use disorders (SUDs) (Handelsman et al., 2000; Speranza et al., 2004; Cleland et al., 2005; De Rick and Vanheule, 2006; Oyefeso et al., 2008; Lindsay and Ciarrochi, 2009; Thorberg et al., 2009; Torrado et al., 2013; Nehra et al., 2014). When considering alexithymia as a categorical variable, while rates in the general adult population range between 6 and 17% (Hintikka et al., 2001; Kokkonen et al., 2001; Franz et al., 2008), adults with SUDs—both abstinent and undergoing treatment—show higher percentages. Despite a first overestimation of 78% (Rybakowski et al., 1988), the prevalence is typically estimated in the range of 43.5 to 67% (Taylor et al., 1990; Haviland et al., 1994; Farges et al., 2004; Speranza et al., 2004; Oyefeso et al., 2008; Lindsay and Ciarrochi, 2009; Thorberg et al., 2009), even though a recent review asserts that it is about 30–49% (Cruise, 2017). Differences in alexithymia rates can be explained by looking at the assessment methods applied and sample characteristics, including the severity of the disorder, the type of treatment (outpatient or inpatient), and the substance being abused (alcohol, opioids, etc.,). Also, when alexithymia is measured as a continuous variable, individuals with SUDs show higher alexithymia traits (Handelsman et al., 2000; Cleland et al., 2005; Ghalehban and Besharat, 2011; Lyvers et al., 2012; Torrado et al., 2013; Nehra et al., 2014). Difficulties in identifying and expressing emotions are also related to increased drug use among adolescents (Trinidad and Johnson, 2002), and using cut-off scores, alexithymia prevalence among young substance abusers (aged 14–25) is noteworthy, ranging from 35 to 43% (Troisi et al., 1998; Farges et al., 2004; Dorard et al., 2008a,b, 2017; Parolin et al., 2017). Studies have used the self-report TAS-20 and almost exclusively address outpatient youth with cannabis use disorders (including both abuse and dependence), with only two exceptions. The study by Farges et al. (2004) did not specify the substance, and in the study by Parolin et al. (2017), a majority of the inpatient participants were opioid dependent. Despite rates among young substance users seeming higher than that among the general population of youth (Säkkinen et al., 2007), when young substance users and controls were compared, the difference in prevalence did not reach statistical significance (Dorard et al., 2008b).

The relationship between alexithymia and addiction is supported by a significant positive association between alexithymic traits and craving, the severity of the disorders and related difficulties (Cleland et al., 2005; Thorberg et al., 2010, 2011a,b). It has been hypothesized that alexithymia may be a vulnerability factor that predates SUDs (Taylor et al., 1997; De Rick and Vanheule, 2006; de Timary et al., 2008). As suggested by Taylor et al. (1997), the role of alexithymia as risk factor for SUDs may be explained by taking into account inherent aspects of the construct (such as immature self-awareness and scarce cognitive regulation of one's emotions). Alternatively, it can be the result of interactions with other risk factors, such as drug expectations, negative affectivity, insecure attachment, executive function, and personality disorders (Pinard et al., 1996; Lumley, 2000; Thorberg et al., 2009; Lyvers et al., 2012; De Carli et al., 2016). Despite some data on clinical (Cecero and Holmstrom, 1997; Uzun, 2003) and non-clinical populations (Kauhanen et al., 1992; Bruce et al., 2012) confirming the theoretical assumption suggesting that alexithymia is a risk factor in the genesis of SUDs, questions remain, and empirical evidence is scarce and non-univocal, as reported by Thorberg et al. (2009) in a review study. Moreover, alexithymia may be a predisposition factor for psychiatric disorders others than drug addiction (Taylor et al., 1997), thus calling the specificity of the association with SUDs into question. Thus, the relationship between alexithymia and drug dependence remains quite unclear (Teixeira, 2017); alexithymia might be a consequence or correlate of the drug disorder (Thorberg et al., 2009).

In alexithymia research, the TAS-20 is the most widely used and studied measure (Taylor et al., 2003; Meganck et al., 2008). Several studies have adopted the TAS-20 to investigate alexithymia in patients with SUDs (Haviland et al., 1988c, 1994; Taylor et al., 1990; Haviland, 1996), reporting rates of 42–50%, thus higher than non-clinical (4–18%) and psychiatric groups (12–33%) (Handelsman et al., 2000; Taylor, 2000). The TAS-20 can discriminate well between psychiatric young patients and non-clinical youth (Kooiman et al., 2002; Marchesi et al., 2014).

Despite its worldwide use in research and clinical practice, the TAS-20 has been criticized for some shortcomings: a critical review of the literature revealed the insufficient reliability of its third subscale (“Externally oriented thinking”), showed the presence of different factor structures in various patient samples and underlined a lack of studies on its criterion validity (Kooiman et al., 2002). As a matter of fact, there are significant relationships between alexithymia, as measured by the TAS-20, and negative affects, depression, and anxiety in both non-clinical (Honkalampi et al., 2010; Deno et al., 2011) and clinical samples (Marchesi et al., 2000; Gatta et al., 2016), including patients with SUDs (Haviland et al., 1988a,b, 1991, 1994; Taylor et al., 1990; Farges et al., 2004; de Haan et al., 2011, 2012a; Morie et al., 2015). In order to examine if the TAS-20 measures the broader construct of negative affects rather than identifying alexithymia itself in clinical groups, Marchesi et al. (2014) compared patients with different diagnoses (major depression, panic disorder, eating disorder, and SUD) to controls. The results indicated that all clinical groups showed higher TAS-20 scores than the controls but not when controlling for anxiety and depression, suggesting that alexithymia as measured by the TAS-20 may have an issue with discriminant validity.

This leads to a more general limitation concerning the assessment of alexithymia with self-report measures, which can be called into question, since they require respondents to report on their psychological states, yet alexithymic individuals lack this capacity by definition (Lane et al., 1997; Lumley, 2000; Waller and Scheidt, 2004). In the specific case of SUDs, substance abusers self-reported higher alexithymia on the TAS-20 than controls and patients with other psychiatric disorders, but their actual performance on a task that required them to identify and describe feelings was not significantly different (Lindsay and Ciarrochi, 2009). As the authors of the instrument acknowledged (Taylor et al., 1997), a multi-method approach is recommended to assess alexithymia: the TAS-20 could be used in combination with other-report instruments (Kooiman et al., 2002). Unfortunately, to date, few studies have compared different measures, especially in the field of addiction. The Observer Alexithymia Scale (OAS; Haviland et al., 2000) represents an alternative assessment measure. The OAS has been used in studies on substance abusers, both adults and adolescents, together with the TAS-20 (Dorard et al., 2008a; Thorberg et al., 2010, 2013; Parolin et al., 2017). It demonstrated adequate psychometric properties and rather low correlations with the TAS-20 total scores and subscales, indicating a lack of correspondence between the two measures (Dorard et al., 2008a).

### Alexithymia as predictor of treatment outcomes

Since alexithymia is a well-recognized and clinically relevant concept, studies have examined whether alexithymic traits may have implications for how drug-dependent patients respond to treatment, but the empirical evidence is non-univocal. Alexithymia (encompassing low self-awareness and interest in introspective activities, scarce empathy and emotion regulation, high negative affectivity and impulsivity and non-optimal coping strategies) may impede treatment and facilitate the use of substances in case of heightened distress (Bagby et al., 1993; Parker et al., 1998; Oyefeso et al., 2008; Shishido et al., 2013). Some evidence has attested that alexithymia may interfere with treatment success. As regards relapse, a cross-sectional investigation on outpatients with alcohol use disorder (Ziółkowski et al., 1995) found significant differences in total scores and alexithymia rates between long abstainers (> 1 year) (33% of alexithymics) and short abstainers ( < 1 year) (63% of alexithymics); stepwise multiple linear regression analysis indicated that the overall TAS-20 score accounted for 20% of the variation in abstinence. Similarly, in a cohort of outpatients with alcohol use disorders, Loas et al. (1997) found significantly higher levels of alexithymia at treatment intake among outpatients who relapsed at 15-month follow-up, even after controlling for depression. The TAS-20 factor accounted for 17% of the variance in abstinence, indicating that alexithymia can predict higher risk of relapse. Alexithymia may also predict treatment engagement, in terms of session attendance and working alliance (drug-dependent outpatients who were higher in alexithymia attended fewer sessions and formed weaker alliances) (Cleland et al., 2005). In studies on inpatients with alcohol use disorder (de Haan et al., 2012b), baseline alexithymia showed no relation to abstinence, time in treatment or changes in disorder severity at 1-year follow-up. Similarly, as concerns SUDs (de Haan et al., 2011), alexithymia (measured as both a continuous and a categorical variable) was not related to abstinence, and high-scoring alexithymics did not differ from low-scoring alexithymics in mean time in treatment or dropout rates (50 vs. 43%). A prospective study on alcoholics (Junghanns et al., 2005) found that alexithymia scores were not associated with the risk of relapse at 6-week follow-up. A recent study confirmed that alexithymia was not strongly associated with treatment adherence or retention in an 8-week randomized clinical trial (Morie et al., 2015). Thus, empirical evidence on the relationship between alexithymia and treatment outcome in SUDs is limited and non-univocal. Substance use treatment is hindered by high rates of relapse (60–70%) (Bradizza et al., 2006) and premature termination, to the extent that it is more common for a patient to drop out of addiction treatment than to complete the treatment (Stark, 1992; Brorson et al., 2013). On the contrary, completion of addiction treatment is one of the most consistent factors associated with a favorable treatment outcome (Hser et al., 2004). This implies the importance of identifying predictors of treatment retention and adherence.

Alexithymia has been recognized as being associated with several psychiatric disorders, including chronic pain. The mediation of pain intensity by prescription painkiller use suggests a process in which more intense pain leads to more frequent use of stronger (prescription) painkillers, which increases the risk of dependence (Elander et al., 2014). The self-medicating hypothesis proposes that individuals use substances to cope with negative affects (Ghalehban and Besharat, 2011). Because of their cognitive inability to identify their emotions, alexithymic individuals may use drugs to regulate their emotions and alleviate stress (Shorin, 1998). Thus, it is difficult to determine whether alexithymia is a specific characteristic of SUDs. These doubts increase in light of the controversy over how to assess the construct to discriminate between primary and secondary alexithymia and related outcomes. Since some evidence has raised concerns on the TAS-20's discriminant validity, a multimethod assessment could spread light on this debate. In addition, little research has addressed this issue in adolescents. Finally, it is not clear whether alexithymia can be considered a risk factor for negative treatment outcomes.

### Objectives

The aim of the present study is to investigate alexithymia in substance-dependent young adults, focusing on some methodological issues. In particular, we are interested in examining whether the available and commonly used assessment measures (self-report and observational) are suitable for evaluating alexithymia in SUD populations. We examine this issue by investigating two objectives: (a) the specificity of alexithymia in drug-dependent inpatients, compared to healthy individuals and patients with psychiatric disorders, and (b) the predictivity of alexithymia in determining treatment outcomes in terms of relapses, drop-outs from treatment and rates of relapse per month of treatment. To fulfill these aims, two studies were conducted, referred to as Study 1 (a) and Study 2 (b).

Study 1 investigates whether young adults diagnosed with SUDs differ from referred psychiatric outpatients and controls in terms of alexithymia levels, as measured by the TAS-20. Based on previous literature, we hypothesized that drug-dependent and referred youths would show higher levels of alexithymia than controls.

Study 2 focuses on a group of inpatients with SUDs and addresses two questions. First, we investigated whether self-report and observational measures differ in their evaluation of alexithymia. According to previous studies, our hypothesis proposes a lack of correspondence between self-report and observational measures, in light of the doubts regarding the validity of self-report tools in assessing alexithymia in clinical samples. Second, Study 2 investigated the ability of alexithymia to predict treatment outcomes. Based on the relevance ascribed to alexithymia in clinical practice and recognizing the lack of consensus on the association between alexithymia and SUD treatment outcomes, we were interested in the predictive role of observed and self-reported alexithymia (at the baseline) for treatment response 1 year after admission, in terms of relapses, dropouts from treatment and rates of relapse per month of treatment. We hypothesized that alexithymia (implying poor introspective capacity, high negative affectivity and non-optimal coping) can predict higher risk of relapse and dropout from treatment. A negative relationship with treatment success would support the need to specifically address alexithymia and adjust treatment protocols.

## Study 1

### Materials and methods

#### Participants

Study 1 involved 90 late adolescents, aged 17–21, who comprised two clinical groups and a comparison group Table 1. The clinical groups included 30 inpatients diagnosed with SUDs and admitted to a residential treatment facility (SUD group) and 30 late adolescents referred to an outpatient neuropsychiatric unit (clinically referred group).

**Table 1:** Descriptive characteristics of each group.

	**Sud group**		**Clinical groups**		**Healty group**	
	***M***	***SD***	***M***	***SD***	***M***	***SD***
Age	19.40	1.25	17.93	1.44	18.70	1.24
	***N*** **(%)**		***N*** (%)		***N*** **(%)**	
Male	16 (53)		16 (47)		12 (40)	
Female	14 (46)		14 (53)		18 (60)	
Poli-abusers	(90)					
Heroin as primary drug of abuse	(63)					

The SUD group included 30 young inpatients admitted to a therapeutic community for SUDs (Villa Renata, Comunità di Venezia, Venice, Italy) and met the following inclusion criteria: (a) diagnosed with SUD according to DSM-5 (APA, 2013) criteria; (b) referred and admitted to the residential treatment community for less than 3 months; and (c) age ranging from 17 to 21 years.

A cohort of 30 patients referred to the Mental Health Public Service (SCIAF ULSS 6, Padua, Italy) for psychopathological problems was recruited. The inclusion criteria were: age between 17 and 21 years, a diagnosis classification of “behavioral and emotional disorders with onset usually occurring in childhood and adolescence” (F90-F98) or “affective [mood] disorders” (F30-F39) (according to ICD-10; World Health Organization, 2016), and no mental delay (QI > 70, according to Wechsler, 2003).

The comparison group included 30 healthy young adults, recruited in high schools near Venice, Italy. The main selection criteria used in the data collection were: (a) the absence of psychiatric disorder diagnosis; (b) absence of current drug use; and (c) age between 17 and 21 years.

#### Procedure and instruments

This first study was carried out in accordance with the recommendations of the Code of Ethics approved by the General Assembly of the Italian Association as well as the Ethical Committee of University of Padua (protocol reference number: 2038). All of the subjects provided written informed consent (parental, in the case of minors) to participate to the study.

All of the participants completed the TAS-20 and the SCL-90-R; for the two clinical groups, administration occurred at treatment intake, as part of an assessment protocol.

- *The 20-item Toronto Alexithymia Scale* (TAS-20; Bressi et al., 1996). Developed by Bagby et al. (1994a,b), the TAS-20 is a self-report scale made of 20 items that must be rated from 1 (*strongly disagree*) to 5 (*strongly agree*); the sum of the items generates a total score and scores for three subscales. The scale has a three-interrelated-factor solution: difficulty identifying feelings (F1), difficulty describing feelings (F2), and externally oriented thinking (F3). Although predominantly used as a dimensional construct, the total score can be compared to cut-off scores that categorize respondents into alexithymic (≥ 60), borderline/intermediate (≤ 51 and ≥ 60) and non-alexithymic. The scale was evaluated as a reliable and valid measure in non-clinical and clinical samples (Parker et al., 1993, 2003; Bagby et al., 1994a,b; Bressi et al., 1996; Taylor et al., 2003), even though the TAS-20 has shown some psychometric shortcomings (Kooiman et al., 2002). The reliability and factor solution of the TAS-20 in samples of substance abusers have been tested and shown sufficiently good results, with the only exception being the Externally Oriented Thinking subscale (Haviland et al., 1988c; Cleland et al., 2005). Moreover, an Italian study conducted on adolescents (La Ferlita et al., 2007) only partially replicated the original factor structure of the TAS-20 and showed higher levels of alexithymic traits in comparison to adults.
- *The Symptom Checklist-90-Revised* (SCL-90-R; Derogatis, 1994). The SCL-90-R is a self-report measure assessing 90 clinical symptoms on a 5-point Likert scale, ranging from 0 (*not at all*) to 4 (*extremely*). The symptoms are factored into nine psychiatric dimensions (depression, anxiety, somatization, obsessive-compulsive behavior, interpersonal sensitivity, hostility, phobic anxiety, psychoticism, and paranoid ideation) plus altered appetite and disturbed sleep. The instrument provides three global scores: the global stress index (GSI), the positive symptom total (PST) and the positive symptom distress index (PSDI). Both the original version and the Italian translation (Sarno et al., 2011) show adequate psychometric properties (Derogatis, 2011). The present study is based on the SCL-90-R, since it can distinguish between clinical and non-clinical individuals via the cut-off score of the GSI.

### Plan of analysis

First, the TAS-20 scale scores were compared between the substance-dependent, referred clinical sample, and non-clinical control groups. One ANOVA model was computed for each TAS-20 subscale, controlling for participants' gender and age. Then, partial correlations between alexithymia and depressive symptomatology were computed, controlling for gender and age.

### Results

**Figure 1:**
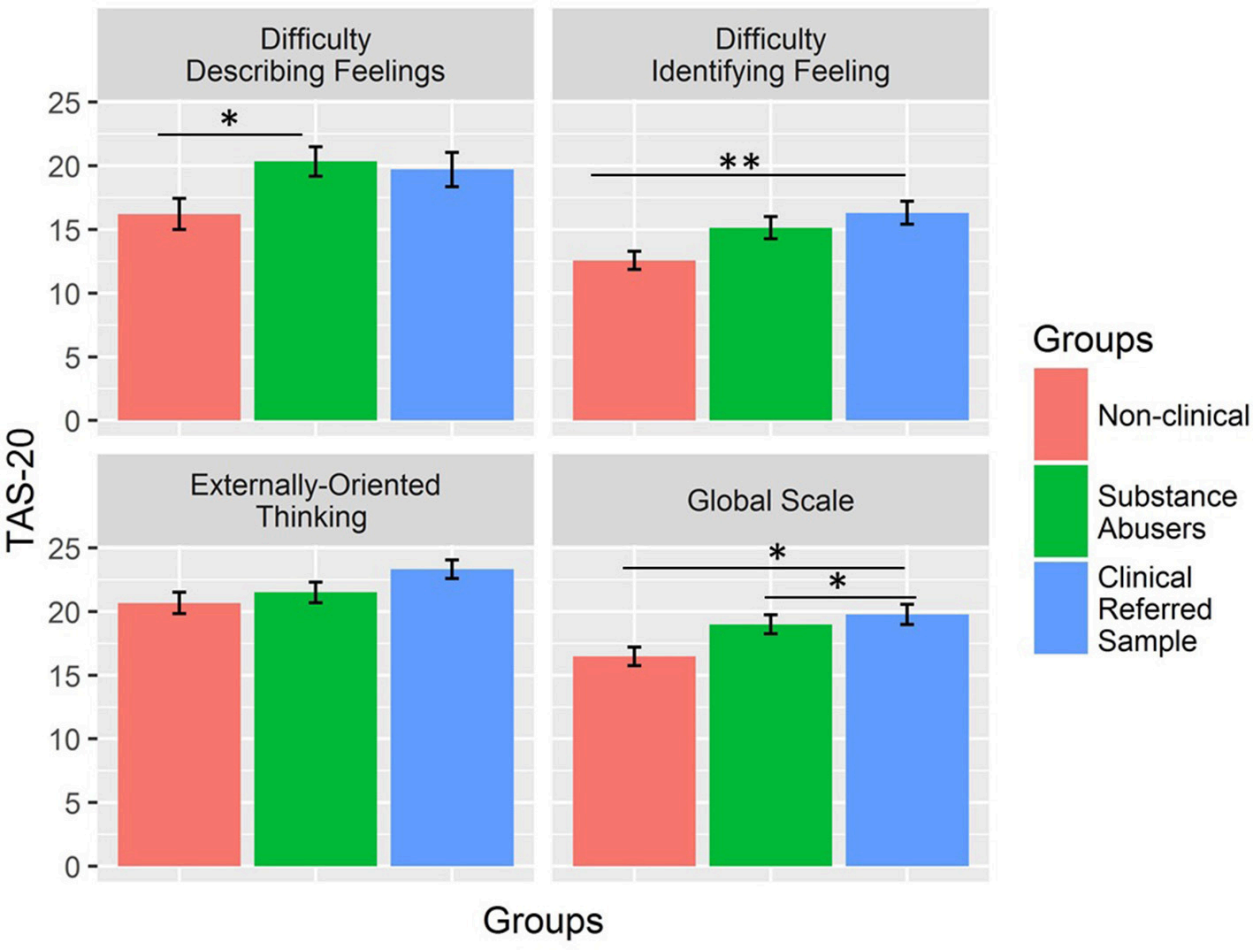
TAS-20 differences between groups.*p < 0.05; **p < 0.01.

Figure 1 shows the differences in TAS-20 scores between the groups. Controlling for age and gender, the effect of group was significant for Difficulty in Describing Feelings [*F*_(2,85)_ = 3.19, *p* = 0.046, η_*p*_^2^ = 0.08], Difficulty Identifying Feelings [*F*_(2,85)_ = 5.17, *p* = 0.008, η_*p*_^2^ = .11], and Global Scale [*F*_(2,85)_ = 5.14, *p* = 0.008, η_*p*_^2^ = 0.11] but not for Externally Oriented Thinking [*F*_(2,85)_ = 2.84, *p* = 0.06, η_*p*_^2^ = 0.05]. Two out of three significant results remained unaltered after Bonferroni correction for multiple comparisons (significance threshold, *p* < 0.013). The Difficulty in Describing Feelings scale did not survive the correction for multiple comparison. Post hoc comparison (Tukey contrasts) showed that the substance-dependent and non-clinical control groups differed on the Difficulty Describing Feelings scale (*b* = 4.13, 95% CI [0.58; 7.69], *p* = 0.02), but there was no difference between the substance-dependent and referred patient groups (*b* = −0.63, 95% CI [−4.19; 2.92], *p* = 0.91) as well as between non clinical controls and referred patients (*b* = 3.50, 95% CI [−0.06; 7.06], *p* = 0.06). For Difficulty Identifying Feeling, only the referred patient group differed from the control group (*b* = 3.73, 95% CI [1.06; 6.41], *p* = 0.004) because there was no difference between the substance-dependent group and both the control (*b* = 2.57, 95% CI [−0.11; 5.24], *p* = 0.06) and referred patient groups (*b* = 1.17, 95% CI [−1.51; 3.84], *p* = 0.55). Finally, for the Global Scale, we found significant differences when comparing the substance-dependent (*b* = 2.51, 95% CI 0.21; 4.81], *p* = 0.03) and referred patient groups (*b* = 3.29, 95% CI 0.99; 5.59], *p* = 0.003) with the control groups, but no differences between them (*b* = 0.78, 95% CI [−1.52; 3.08], *p* = 0.70).

In the global sample, the partial correlation between alexithymia and depression, as measured by the SCL-90-R and controlling for gender and age, was *r*_(88)_ = 0.45, *p* < 0.001. The correlation was essentially the same in the substance abusers group, *r*_(28)_ = 0.44, *p* = 0.01.

## Study 2

### Materials and methods

#### Participants

A total of 55 inpatients with SUD were involved in Study 2; in addition to those who participated to Study 1 (*N* = 30), 25 additional participants were included.

Inpatients were recruited from a therapeutic community (Villa Renata, Comunità di Venezia, Venice, Italy). The therapeutic community treatment model (De Leon et al., 2015), is based on a long-term residential and intensive approach that combines therapeutic and educational activities. Inpatients attend daily occupational, house-service, and recreational activities together with staff members, who offer monitoring and support to foster self-help learning. Weekly individual and group psychotherapy is provided, with the primary goal of changing the negative patterns of behavior, thinking, and feeling that predispose the individual to drug use as well as developing interpersonal skills and psychological wellbeing.

The participants (described in Table 2) fulfilled the following inclusion criteria: (a) met the DSM-5 (APA, 2013) criteria for SUD; (b) referred and admitted to the residential treatment community for less than 3 months; and (c) age ranging from 17 to 24 years. At recruitment, the participants had been abstinent for 2.83 months on average. Additionally, 46% overdosed from one to three times in the past, and 21% had a drug-related illness (hepatitis C). In relation to treatment, 40% of participants had previously attended an inpatient treatment but had not concluded it. At 1 year after admission, 49% of the participants dropped out from treatment, while 59% relapsed during the 12 months of treatment. These data are in line with previous studies, reporting dropout rates of 17–57% for residential treatment (Brorson et al., 2013) and relapse rates of 40–60% (McLellan et al., 2000).

**Table 2:** Descriptive characteristics of SUD group.

**INPATIENTS SUD GROUP**			
**Age**	**Age**	***M***	***SD***
		21.10	2.15
		***N*** **(%)**	
Gender	Male	30 (54%)	
	Female	25 (45%)	
SES	Not attained an upper secondary educational qualification	33 (60%)	
	Unemployed	35 (63%)	
Past history	Had one or both parents presenting a past or current Substance Use Disorder	24 (43%)	
	Experienced maltreatment, sexual or physical abuse during childhood	34 (61%)	
Comorbidities	Psychiatric illness	16 (29%)	
Poly-drug use	Poly-drug use	47 (86%)	
Primary substance of abuse	Different synthetic drugs	46 (83%)	
	Cocaine	10 (18%)	
	Heroin	38 (69%)	
	Use of non-prescribed drugs	29 (53 %)	

#### Procedure and instruments

This second study was carried out in accordance with the recommendations of the Code of Ethics approved by the General Assembly of the Italian Association as well as the Ethical Committee of University of Padua (protocol reference number: 2038). All patients provided written informed consent (parental, in the case of minors) to participate to the study.

The participants of Study 2 completed the TAS-20 at admission, and their individual treating psychologists completed the OAS after 8 to 12 weeks of treatment. After 1 year of residential treatment, or after dropout, the number of relapses was reported from the community registers.

- *The Observer Alexithymia Scale* (OAS; Haviland et al., 2000). The OAS is a 33-item observational scale to be completed by a subject's relative or acquaintance. The instrument was developed by asking clinicians to describe the prototypical characteristics of an alexithymic person. Items are rated on a 4-point scale and cover five alexithymic features: distant (being unskilled in intrapersonal and interpersonal issues), uninsightful, somatizing, humorless, and rigid. The reliability and validity of the OAS have been tested in both non-clinical (Haviland et al., 2000) and clinical samples (Haviland et al., 2001; Thorberg et al., 2010). Adequate internal consistency, test-retest reliability and factorial validity emerged; moreover, total OAS scores differed significantly between the clinical and non-clinical groups (Haviland et al., 2001). Despite these results and its clinical utility, the OAS presents some limitations: some researchers (Meganck et al., 2010) have questioned its validity because of insufficient interrater reliability and problematic criterion validity (it seems that the OAS is based on a broader definition of alexithymia than the original one, including some characteristics that are correlates of alexithymia rather than constitutive dimensions).

### Plan of analysis

In different regression models controlling for gender and age, both self-report and observational measures of alexithymia were used as predictors for the number of relapses, number of dropouts from treatment, and the rates of relapse per month of treatment. We used the ratio between the number of relapses to months of treatment due to the high incidence of dropouts, which made the absolute number of relapses a potentially biased effect depending on the length of the hospitalization. Next, gender was added as a possible moderator of the association between alexithymia and outcome measures.

### Results

**Figure 2:**
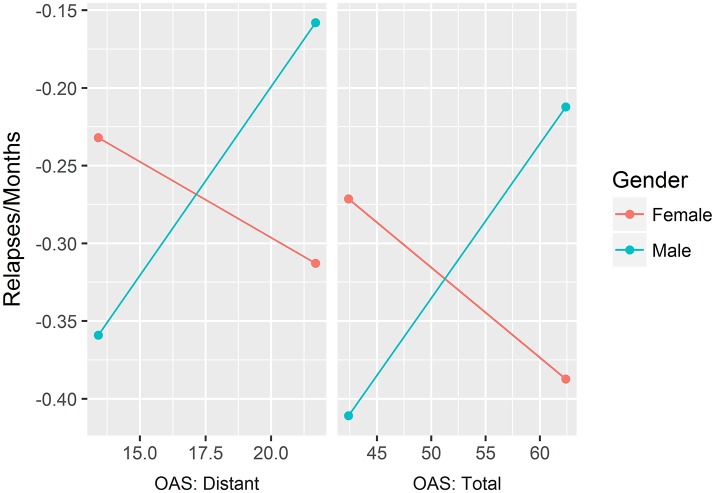
Simple slope analyses of the effects of 2 OAS scales (i.e, Distant and Total) on the rate of relapses per month.

Table 3 shows the partial correlations between observed and self-reported alexithymia after controlling for gender and age. Table 4 presents all of the linear and logistic regression models used to test the role of alexithymia (both self-reported and observed) in predicting relapses and dropouts. For each model, the interaction effect gender^*^alexithymia is also provided. Since the interactions between gender and two scales of the OAS were significant in predicting the rate of dropouts per months of treatment, we computed simple slope analyses to explore these results, which are plotted in Figure 2. The effects of the OAS total score scale were positive and significant for males [*b* = 0.01, SE = 0.005, *t*_(44)_ = 2.15, *p* = 0.04] but not significant for females [*b* = −0.006, SE = 0.003, *t*_(44)_ = −1.73, *p* = 0.09]. Indeed, the same effect was found for the Distant scale, which was significant and positive for males [*b* = 0.03, SE = 0.01, *t*_(44)_ = 2.60, *p* = 0.01] but not significant for females [*b* = −0.01, SE = 0.01, *t*_(44)_ = −1.04, *p* = 0.30].

**Table 3:** Partial correlations between self-reported and observed alexithymia measures controlling for gender and age.

	**1**	**2**	**3**	**4**	**5**	**6**	**7**	**8**	**9**	**10**
Difficulty identifying feelings (F1)										
Difficulty describing feelings (F2)	0.58^***^									
Externally Oriented Thinking (F3)	0.30^*^	0.38^***^								
TAS 20 Total	0.86^***^	0.83^***^	0.64^***^							
Distant	−0.05	−0.09	0.14	−0.02						
Uninsightful	0.24^†^	0.07	−0.01	0.15	0.37^**^					
Somatiazing	−0.01	−0.15	−0.07	−0.09	0.14	0.18				
Humorless	−0.01	0.13	0.26^†^	0.13	0.31^*^	0.43^***^	0.19			
Rigid	−0.33^*^	−0.11	−0.15	−0.27^*^	0.24^†^	0.26^*^	0.17	0.32^*^		
OAS Total	−0.01	−0.06	0.04	−0.02	0.72^***^	0.75^***^	0.5^***^	0.61^***^	0.57^***^	

*** p < 0.001;

** p < 0.01;

* p < 0.05;

† *p < 0.1*.

**Table 4:** Regression models to test alexithymia predictive ability.

	**Linear regressions**				**Logistic regressions**				**Linear regressions**			
	**Relapses**		**Relapses**		**Drop-out**		**Drop-out**		**Relapses/Months**		**Relapses/Months**	
	***b***	***t***	***b***	***t***	***b***	**z val**	***b***	**z val**	***b***	***t***	***b***	***t***
OAS: Distant	0.02	0.60	0.10	1.83^†^	−0.04	−0.50	0.07	0.70	0.01	1.05	0.02	2.54^**^
Gender	0.11	0.33	0.08	0.24	−0.43	−0.74	−0.49	−0.82	−0.01	−0.19	−0.02	−0.33
Age	0.10	1.36	0.11	1.48	0.06	0.42	0.07	0.50	0.02	1.34	0.02	1.51
Distant ^*^Gender			−0.16	−2.05^*^			−0.25	−1.57			−0.03	−2.56^**^
*R*^2^	0.05		0.13						0.07		0.19^*^	
Model Confrontation	*F*_(1, 50)_ = 4.21, *p* < 0.05				$\chi_{\left(1\right)}^{2}$ = 2.69, *p* > 0.05				*F*_(1, 50)_ = 6.57, *p* < 0.01			
OAS: Unisightful	−0.01	−0.36	0.06	0.91	−0.05	−0.67	−0.06	−0.56	0.00	−0.30	0.01	1.17
Gender	0.10	0.30	0.14	0.43	−0.37	−0.64	−0.38	−0.66	−0.02	−0.26	−0.01	−0.12
Age	0.11	1.39	0.12	1.61	0.05	0.37	0.05	0.34	0.02	1.38	0.02	1.66^†^
Unisightful ^*^Gender			−0.12	−1.48			0.03	0.18			−0.03	−1.77^†^
*R*^2^	0.05		0.09						0.04		0.11	
Model Confrontation	*F*_(1, 50_) = 2.18, *p* > 0.05				$\chi_{\left(1\right)}^{2}$ = 0.03, *p* > 0.05				*F*_(1, 50)_ = 3.14, *p* = 0.08			
OAS: Somatizing	−0.02	−0.33	0.17	1.54	−0.01	−0.05	0.00	−0.02	−0.01	−0.65	0.02	0.86
Gender	0.15	0.39	−0.01	−0.02	−0.39	−0.60	−0.39	−0.59	0.00	0.04	−0.02	−0.26
Age	0.11	1.39	0.10	1.32	0.05	0.38	0.05	0.38	0.02	1.38	0.02	1.31
SOM^*^Gender			−0.27	−2.03^*^			0.00	−0.01			−0.03	−1.42
*R*^2^	0.05		0.13						0.05		0.09	
Model Confrontation	*F*_(1, 50)_ = 4.11, *p* < 0.05				$\chi_{\left(1\right)}^{2}$ = 0.01, *p* > 0.05				*F*_(1, 50)_ = 2.03, *p* > 0.05			
OAS: Humorless	0.04	0.37	0.19	1.51	0.02	0.13	0.18	0.78	0.00	0.15	0.02	1.04
Gender	0.08	0.24	0.14	0.42	−0.41	−0.70	−0.36	−0.62	−0.02	−0.30	−0.01	−0.17
Age	0.11	1.43	0.13	1.76^†^	0.05	0.40	0.08	0.56	0.02	1.39	0.02	1.63
Humorless ^*^Gender			−0.43	−2.05^*^			−0.46	−1.15			−0.06	−1.57
*R*^2^	0.05		0.13						0.04		0.09	
Model Confrontation	*F*_(1, 50)_ = 4.22, *p* < 0.05				$\chi_{\left(1\right)}^{2}$ = 1.38, *p* > 0.05				*F*_(1, 50)_ = 2.48, *p* > 0.05			
OAS: Rigid	−0.09	−1.43	−0.05	−0.65	−0.15	−1.23	0.00	0.03	−0.01	−1.08	0.00	−0.27
Gender	0.05	0.15	0.04	0.12	−0.49	−0.83	−0.60	−0.96	−0.02	−0.39	−0.02	−0.42
Age	0.14	1.75^†^	0.14	1.80^†^	0.10	0.71	0.14	0.94	0.02	1.63	0.02	1.72^†^
RIG^*^Gender			−0.07	−0.60			−0.37	−1.47			−0.02	−0.79
*R^2^*	0.08		0.09						0.07		0.08	
Model Confrontation	*F*_(1, 50)_ = 0.36, *p* > 0.05				$\chi_{\left(1\right)}^{2}$ = 2.41, *p* > 0.05				*F*_(1, 50)_ = 0.62, *p* > 0.05			
OAS: Total	0.00	−0.30	0.04	1.77^†^	−0.02	−0.79	0.01	0.30	0.01	−0.14	0.01	2.08^*^
Gender	0.10	0.31	0.10	0.33	−0.35	−0.60	−0.36	−0.60	−0.02	−0.27	−0.02	−0.29
Age	0.11	1.41	0.13	1.74^†^	0.06	0.44	0.07	0.54	0.02	1.39	0.02	1.77^†^
OAS_tot^*^Gender			−0.08	−2.52^*^			−0.06	−1.02			−0.02	−2.78^**^
*R^2^*	0.04		0.17^†^						0.04		0.19^†^	
Model Confrontation	*F*_(1, 50)_ = 6.37, *p* > 0.05				$\chi_{\left(1\right)}^{2}$ = 1.05, *p* > 0.05				*F*_(1, 50)_ = 7.75, *p* > 0.05			
TAS: Difficulty identifying feelings	−0.02	−0.75	−0.04	−1.07	−0.02	−0.38	−0.07	−0.99	0.00	−0.47	−0.01	−0.87
Gender	0.14	0.42	0.11	0.33	−0.36	−0.61	−0.44	−0.73	−0.01	−0.19	−0.02	−0.27
Age	0.10	1.31	0.11	1.44	0.05	0.34	0.08	0.57	0.02	1.33	0.02	1.46
TAS^*^Gender			0.04	0.77			0.10	1.02			0.01	0.76
*R^2^*	0.05		0.07						0.05		0.06	
Model Confrontation	*F*_(1, 50)_ = 0.59, *p* > 0.05				$\chi_{\left(1\right)}^{2}$ = 1.08, *p* > 0.05				*F*_(1, 50)_ = 0.58, *p* > 0.05			
TAS: Difficulty communicating feelings	−0.03	−0.84	−0.04	−0.83	−0.03	−0.57	−0.08	−0.89	−0.01	−1.99^*^	−0.02	−2.31^*^
Gender	0.19	0.54	0.17	0.49	−0.29	−0.47	−0.35	−0.56	0.02	0.38	0.01	0.23
Age	0.1	1.32	0.1	1.34	0.05	0.33	0.06	0.43	0.02	1.26	0.02	1.41
F2_tot^*^Gender			0.02	0.32			0.09	0.07			0.01	1.22
*R^2^*	0.06		0.06						0.12		0.15	
Model Confrontation	*F*_(1, 50)_ = 0.1, *p* > 0.05				$\chi_{\left(1\right)}^{2}$ = 0.50, *p* > 0.05				*F*_(1, 50)_ = 1.50, *p* > 0.05			
TAS: Externally oriented thinking	−0.03	−0.78	−0.04	−0.68	0.03	0.50	0.00	0.02	−0.01	−1.01	−0.01	−1.36
Gender	0.08	0.26	0.08	0.25	−0.40	−0.69	−0.40	−0.69	−0.02	−0.32	−0.02	−0.32
Age	0.09	1.12	0.09	1.12	0.07	0.51	0.08	0.58	0.01	1.05	0.02	1.16
TAS^*^Gender			0.01	0.15			0.07	0.54			0.01	0.91
*R^2^*	0.06		0.06						0.06		0.08	
Model Confrontation	*F*_(1, 50)_ = 0.02, *p* > 0.05				$\chi_{\left(1\right)}^{2}$ = 0.30, *p* > 0.05				*F*_(1, 50)_ = 0.82, *p* > 0.05			
TAS: Total	−0.01	−1.00	−0.03	−1.24	−0.25	0.80	−0.04	−0.95	0.00	−1.37	−0.01	−2.06
Gender	0.17	0.50	0.15	0.45	−0.62	0.53	−0.42	−0.70	0.00	0.03	0.00	−0.06
Age	0.09	1.19	0.10	1.30	0.33	0.74	0.07	0.51	0.01	1.12	0.02	1.38
TAS^*^Gender			0.02	0.78			0.05	1.05			0.01	1.52
*R2*	0.06		0.08						0.08		0.13	
Model Confrontation	*F*_(1, 50)_ = 0.61, *p* > 0.05				$\chi_{\left(1\right)}^{2}$ = 1.05, *p* > 0.05				*F*_(1, 50)_ = 2.30, *p* > 0.05			

^***^p < 0.001;

** p < 0.01;

* p < 0.05;

† *p < 0.1*.

## Discussion

The present study contributes to the current debate on alexithymia, specifically concerning two controversial aspects: its associations with SUDs (i.e., in terms of prevalence and treatment predictivity) and some assessment issues, since the TAS-20 has received criticism, despite its worldwide use and validity.

### Specificity of alexithymia in drug-dependent young adults

The aim of Study 1 was to investigate whether late adolescents diagnosed with SUDs differ from late adolescent referred psychiatric outpatients and controls in levels of alexithymia, as measured by the TAS-20. On the basis of previous literature attesting that the TAS-20 can discriminate well between psychiatric patients and non-clinical youths (Kooiman et al., 2002; Marchesi et al., 2014), we hypothesized that drug-dependent and referred youth would show higher levels of alexithymia than controls. The results indicated that patients in both clinical groups, regardless of their specific disorder, presented higher TAS-20 scores than non-clinical subjects, but the clinical groups did not differ from each other. In considering the lack of difference between the two clinical groups, it is important to acknowledge that SUD populations show high rates of comorbidity and that our study only relied on the SCL-90-R. Our data resemble those of Marchesi et al. (2014), who compared different groups of adult patients (with major depression, panic disorder, eating disorder, and SUDs) and controls. Thus, despite numerous studies suggesting the presence of a specific link between alexithymia and addiction (i.e., as a risk factor), alexithymia might be non-univocally related to SUD nor to other distinct disorders. Instead, it could be associated with the broader concept of psychological distress, regardless of the symptomatological phenomenology. Consistent with this idea, Study 1 highlighted a large correlation between alexithymia and depressive symptoms. A vast body of literature shows that alexithymia is positively related to psychological distress in general and depressive and anxiety symptomatology (Haviland et al., 1991), and this association has also been detected when adopting the TAS-20 in non-clinical (Honkalampi et al., 2010; Deno et al., 2011), clinical (Marchesi et al., 2000) and SUD groups (Haviland et al., 1988a, 1991, 1994; Taylor et al., 1990; de Haan et al., 2011, 2012a; Morie et al., 2015). Recently, empirical support has been given to the notion that alexithymia, as measured by the TAS-20, may represent an issue with discriminant validity (Marchesi et al., 2014) and that the TAS-20 assesses a general psychological distress factor rather than identifying alexithymia itself (Leising et al., 2009).

### Predictivity of self-report and observed alexithymia

Study 2 focused on a group of young adults diagnosed with SUD. First, it examined whether self-report and observational measures differ in the evaluation of alexithymia. Despite a wide consensus on the need to use a multi-method approach to assess alexithymia, such an approach is rarely achieved (Kooiman et al., 2002). In the present sample, clinician-rated alexithymia (by the OAS) was not correlated with self-report alexithymia (by TAS-20); our results are consistent with those of a previous study on adolescent substance-abusers that compared OAS and TAS-20 scores and indicated a lack of correspondence between the scores of the two assessment tools (Dorard et al., 2008a). As a whole, the adequacy of the TAS-20 in assessing alexithymia, as with other self-reports, appears to be questionable. This might particularly be the case for clinical groups characterized by low levels of self-reflective capacity, including such individuals with SUDs.

Contrary to expectations, neither self-reported nor observed alexithymia predicted the number of relapses, retention or dropout from treatment. Our results are in line with those of other empirical works indicating the lack of an association between alexithymia and abstinence, time in treatment and treatment adherence (Junghanns et al., 2005; de Haan et al., 2011, 2012b; Morie et al., 2015).

Different possible explanations can be given for these results. One explanation looks at alexithymia, its stability and its role as a vulnerability factor. As noted by de Haan and colleagues (de Haan et al., 2012a, 2014), alexithymia can be a vulnerability factor for substance use (and thus be reasonably addressed in treatment) only if it is a stable personality trait, but research results are conflicting regarding the stability of alexithymia. Studies support the idea that alexithymia is (at least partially) a state-related phenomenon (de Haan et al., 2012a, 2014). It has been conceived of as a secondary and situational response to negative affectivity, anxiety and depression (Haviland et al., 1988a; Pinard et al., 1996; Taylor et al., 1997; Honkalampi et al., 2000; de Timary et al., 2008; De Carli et al., 2017). Moreover, studies supporting the view of alexithymia as a structural trait in substance-dependent patients consider quite limited periods of time (Keller et al., 1995; Pinard et al., 1996; Rosenblum et al., 2005) or demonstrate its relative but not its absolute stability (de Timary et al., 2008; Thorberg et al., 2016). The state-dependent nature of alexithymia in drug-dependent individuals could explain why alexithymia measured at treatment intake is unrelated to events occurring later (by up to 1 year).

Second, in accordance to other authors (Cleland et al., 2005; Junghanns et al., 2005), the limited predictive capacity of alexithymia may depend on the fact that alexithymia may influence treatment outcomes only when interacting with other factors, such as negative affectivity, anxiety or depressive symptoms. Maybe the availability of more detailed measures of time (including when relapses and treatment dropouts occur), concurrent levels of alexithymia and negative affectivity could help to clarify the relation between alexithymia and treatment indexes.

Third, the lack of association can be explained by the fact that dropout and relapse might depend on different and/or multiple factors, rather than single variables. To date, there is a general lack of consistency in these predictors across studies. A number of factors have shown no or minimal predictivity for treatment retention, including demographic variables (gender, socioeconomic status, employment, and education). The most consistent risk factors for dropout include cognitive deficits, weak treatment alliance, personality disorders, younger age (Brorson et al., 2013), and client motivation (Ball et al., 2006; Palmer et al., 2009). Regarding relapses, investigators have identified some risk factors, like severity of SUD and its sequelae, psychiatric comorbidity, family history of SUDs, stressors, and coping. Importantly, the most reliable predictive models of relapses and dropout take into account a multitude of predictors and their interactions (Bradizza et al., 2006; Moos and Moos, 2006; Brorson et al., 2013; Brecht and Herbeck, 2014).

Finally, methodological issues regarding the assessment measures, sample characteristics, and type of treatment have been pointed to the lack of a strong association between alexithymia and outcomes (Cleland et al., 2005). Regarding treatment, alexithymia may exert a different degree of influence on treatment success by treatment approach (de Haan et al., 2012b; Morie et al., 2015). However, the available results are highly preliminary and limited; thus, conclusions on the relationship between alexithymia and treatment type cannot be drawn.

At an exploratory level, we tested the moderating role of gender on the association between alexithymia and treatment outcomes. The interaction effects between gender and two of the subscales for observed alexithymia were significant. Specifically, the global score and the Distant factor predicted the number of relapses in males but not in females. The effect of the global score seems to be driven by the Distant subscale. This factor describes an avoidant style toward relationships and inner states, and probably toward the treatment alliance too. Due to the explorative nature of the investigation, interpretations should be made extremely carefully. However, future studies could focus on the relevance of avoidant behaviors between males but not females in the quality of treatment outcomes. It is possible that the presence of avoidant strategies is more risky for men than for women, probably because it could be more closely related to impulsive behaviors. Such a finding, even if presented for the first time, has similar results in previous literature, such as a moderating effect of gender in the association between hostility and treatment termination (Petry and Bickel, 2000). The study of moderators of risk factors for treatment dropout is extremely relevant to foster tailored interventions (Brorson et al., 2013) and deserves future attention.

In conclusion, the present study suggests that alexithymia, as measured by the TAS-20, does not distinguish young inpatients with SUDs from referred patients and controls—it only differentiates between clinical and non-clinical groups. As recent empirical studies have proposed, alexithymia might be more associated with negative affectivity or psychological distress, rather than characterizing distinct disorders. These results, together with the lack of correspondence between the TAS-20 and the OAS observational scale, raise doubts on the validity of alexithymia being measured by self-reports. Finally, the limited ability of both observed and self-reported measures in predicting treatment dropout and relapses highlights the need for more complex predictive models in treatment research.

Despite some strengths, such as the multi-method assessment of alexithymia, addressing substance abuse at a specific age (young adulthood) and adopting a wide window of time (1 year), the research shows some limitations. First of all, the relatively small samples involved suggest caution in interpreting the results, particularly the lack of effects of alexithymia in predicting treatment outcomes. In addition, the prediction of outcomes was limited to 1 year of treatment, meaning that no inferences can be made on long-term results of the intervention. In addition, no assessment of symptom severity was available. Furthermore, the lack of systematic biochemical analyses to confirm abstinence/relapses must be acknowledged. Also, with regard to the comparison group of referred patients in Study 1, the participants were not affected by a specific psychiatric disorder but presented heterogeneous diagnoses. Another difference with the SUD group was the condition of being outpatients, instead of inpatients, which is likely to be associated with diminished illness severity. In addition, observed alexithymia was not assessed in the nonclinical or referred samples, since it was not possible to identify observers who were comparable to the therapists for the SUD group. Finally, as already mentioned, the predictivity of alexithymia was analyzed by taking single factors into consideration, rather than multiple variables.

Future studies should try to extend the observational measure of alexithymia to other disorders to explore whether the lack of correlation with the TAS-20 is specific to SUDs. In addition, future perspectives could try to replicate the moderating effect of gender on the association between alexithymia and treatment outcome. Also, not only can observers' ratings of alexithymia improve our understanding of the concept, but so can integration with other fields of study, such as family functioning (Gatta et al., 2017), endocrine functioning (Riem et al., 2017), and brain activity (De Carli et al., 2018).

## Author contributions

MP, MM, PD, PC, MG, and AS have given a substantial contribution to the conception and implementation of the work, taking part to data acquisition, analysis and discussion, drafting, and revising the manuscript. All authors revised and reached an agreement on the final version of the work.

### Conflict of interest statement

The authors declare that the research was conducted in the absence of any commercial or financial relationships that could be construed as a potential conflict of interest.
